# Percutaneous cholecystostomy: a nonsurgical therapeutic option for acute cholecystitis in high-risk and critically ill patients

**DOI:** 10.1590/S1516-31802003000600009

**Published:** 2003-11-06

**Authors:** de Castro Dabus Guilherme, Sérgio San Juan Dertkigil, Jamal Baracat

**Keywords:** Percutaneous cholecystostomy, Criticallyill patients, Acute cholecystitis, Vesícula biliar, Cálculos, Colecistectomia

## Abstract

Percutaneous cholecystostomy offers a potentially important type of therapy for critically ill patients with acute cholecystitis who present high risk when undergoing laparotomy or laparoscopy under general anesthesia. It offers a distinct advantage for these kinds of patients by avoiding the risks of the surgical intervention. Percutaneous cholecystostomy is a safe and effective minimally invasive procedure with a high success rate and low procedurerelated complications. It should be considered not only in temporary management of calculous cholecystitis, but also in definitive treatment in cases of acalculous cholecystitis.

## RESUMO

A colecistostomia percutânea é uma modalidade terapêutica potencialmente importante a ser considerada em pacientes em estado grave com colecistite aguda, que estão sob alto risco ao serem submetidos a laparotomia ou a laparoscopia sob anestesia geral. A colecistostomia pecutânea oferece vantagens neste tipo de paciente por evitar os riscos cirúrgicos da colecistectomia. A colecistostomia percutânea é procedimento seguro, minimamente invasivo e efetivo, com alta taxa de sucesso e com pequeno número de complicações relacionadas ao procedimento. Deve ser considerada não apenas como tratamento temporário da colecistite calculosa, mas também como tratamento definitivo nos casos de colecistite acalculosa.

The occurrence of acute cholecystitis has been reported in up to 18% of trauma patients in intensive care units.^1^ Although cholecystectomy (open or laparoscopic) is generally safe, with an operative mortality rate of 0 to 0.8%, in critically ill patients with high surgical risk the mortality rate rises to a range of 14 to 30%.^2,3^ Such intensive care unit patients are generally debilitated because of serious underlying illness, sepsis of unknown origin or multiorgan disease.^1,2,4^

Surgical cholecystostomy, introduced by Bobbs, was the only available method for gallbladder decompression for more than a century.3 It became an important management option in such patients. Although surgical cholecystostomy can be performed under local anesthesia in the operating room, it has the disadvantage of requiring laparotomy and heavy sedation and may be difficult to perform.^5^

In recent years, percutaneous cholecystostomy has become an alternative nonsurgical therapeutic option for high-risk and critically ill patients with acute cholecystitis.^1-13^ Percutaneous cholecystostomy was first described in 1921, when it was used as a diagnostic test. Ultrasound-guided cholecystostomy with placement of a catheter for therapeutic purposes was reported in 1979, when it was used for treating cholangitis in a case of obstructive jaundice. The first report of percutaneous cholecystostomy for acute cholecystitis was in 1980, when it was performed in a critically ill patient with empyema of the gallbladder.^5^ Since these first descriptions, it has been performed as an alternative to cholecystectomy and surgical cholecystostomy in such patients.^1-13^ Percutaneous cholecystostomy is an efficacious procedure with reported clinical response rates of 56 to 100%, with the lowest of these results from series consisting exclusively or predominantly of critically ill patients.^2-4,14,15^ Percutaneous cholecystostomy can be performed in the intensive care unit under ultrasound guidance, or in the Radiology Department under ultrasound or fluoroscopic guidance, thereby allowing decompression of the inflamed gallbladder and providing a potential route for stone extraction.^5,10^ The gallbladder can be entered by using the Seldinger technique with tract dilatation and catheter placement via guide wire, or by means of the direct trocar technique. The tract chosen depends on the anatomy and whether stone extraction is planned. The transhepatic route is associated with less risk of bile leakage, whereas the transperitoneal route is preferred for stone removal through a larger tract.^10^

A positive response to percutaneous cholecystostomy is defined as when there is defervescence, resolution of symptoms and signs in symptomatic patients, reduction in white blood cell counts to normal or by at least 25%, and a capability for weaning off vasopressors, with all this occurring within 72 hours of the procedure. A negative response is defined as when the patient's clinical course is unaltered after percutaneous cholecystostomy.^1,2,4^ A positive response is more likely to occur in patients who have clinical signs and symptoms that can be related to the gallbladder and upper right quadrant.^2,9^ Patients with ultrasound findings from the gallbladder such as wall thickening, distention, stones, pericholecystic fluid and Murphy's sign have a high chance of a positive response. Pericholecystic fluid and Murphy's sign seem to be the most important radiological findings for predicting a positive response to percutaneous cholecystostomy.^2,4^

The diagnosis of acute cholecystitis in critically ill patients may be difficult. Such patients may not be able to communicate because of complex clinical problems or mechanical ventilation. Unexplained fever, upper right quadrant tenderness and elevated white blood cell counts are the classic findings. The biochemistry is often nonspecifically abnormal, and the cholestatic chemistry that typifies acute cholecystitis is also seen in hepatic dysfunction related to sepsis or multiple organ failure. Ultrasound can demonstrate a gallbladder with distention, wall thickening, sludge, stones, pericholecystic fluid and Murphy's sign. Cholescintigraphy may be helpful but, in patients who have fasted for longer than 14 hours or are undergoing total parenteral nutrition, this may lead to false-positive results.^1,2,4,11^ Some authors have suggested that a lower threshold for performing percutaneous cholecystostomy in patients with sepsis of unknown cause is worthwhile.^4^

Acute acalculous cholecystitis accounts for 5 to 10% of all cases of cholecystitis and typically occurs in patients with acute multisystem disease.^1,11^ If untreated, the short-term mortality may reach 35%.^11^ Patients with acalculous cholecystitis who are treated by means of percutaneous cholecystostomy are unlikely to require further treatment. The likelihood of recurrence is low. In critically ill patients with acute calculous cholecystitis, cholecystostomy does not provide definitive treatment, and the risk of recurrence with the development of acute cholecystitis is significant if the stones are left in-situ. The potentially successful stone removal and dissolution methods include percutaneous cholecystolithotomy, endoscopic retrograde cholangiopancreatography, mechanical basketing of the stones and dissolution using methyl-*tert*-butyl ether^8,9^ After stone removal or dissolution, elective laparoscopic or open cholecystectomy would be strongly considered, since stone recurrence occurs in 8 to 10% of patients per year for the first 3 to 5 years after successful nonsurgical management.^6^

Percutaneous cholecystostomy is a safe and effective minimally invasive procedure with a technical success rate ranging from 98 to 100%. Relief of clinical symptoms varies from 59 to 93% depending on the patient's condition. The complication rate reported for this procedure is 12%.^3,7,12^ Some authors have reported 30-day mortality of 3.1%, but such deaths are considered secondary to severe underlying illness in these patients.^7^ The complications of percutaneous cholecystostomy include vagal reactions, hypotension, bile peritonitis, empyema, catheter dislodgment, bleeding, pneumothorax, pleural effusion and respiratory distress. Vagal reactions and hypotension can be treated with atropine and fluids. The bleeding is probably due to coagulopathy. Respiratory distress is due to pain from attempted catheterization with a thick-walled gallbladder. Catheter dislodgment is reported in 5 to 10% of patients and appears to be the result of a combination of factors including patient confusion, agitation, difficulty in catheter fixation secondary to respiratory movements, and failure to protect the catheter during transportation. Bile peritonitis and death have been reported from bile leakage.^6-8^

In conclusion, the high success rate and the low procedure-related complications of percutaneous cholecystostomy have encouraged and expanded its utilization. In critically ill high-risk patients, it should be considered to be a safe and efficacious means for temporary management of calculous cholecystitis, or possible definitive treatment of patients with acalculous cholecystitis.
